# The attenuated hepatocellular carcinoma-specific Listeria vaccine Lmdd-MPFG prevents tumor occurrence through immune regulation of dendritic cells

**DOI:** 10.18632/oncotarget.3558

**Published:** 2015-03-12

**Authors:** Xin Wan, Ci Cheng, Zhe Lin, Runqiu Jiang, Wei Zhao, Xin Yan, Junwei Tang, Kun Yao, Beicheng Sun, Yun Chen

**Affiliations:** ^1^ Department of Microbiology and Immunology, Nanjing Medical University, Nanjing, Jiangsu Province, China; ^2^ Liver Transplantation Center, The First Affiliated Hospital of Nanjing Medical University, Nanjing, Jiangsu Province, China

**Keywords:** Lmdd-MPFG, Hepatocellular carcinoma, dendritic cells, PRRs

## Abstract

Immunotherapy is a promising treatment for liver cancer. Here, we tested the ability of the attenuated hepatocellular carcinoma-specific Listeria vaccine (Lmdd-MPFG) to treat hepatocellular carcinoma (HCC) in a mouse model. Immunization with the vaccine caused a strong anti-tumor response, especially in mice reinfused with dendritic cells (DCs). In mice that were also administered DCs, tumor suppression was accompanied by the strongest cytotoxic T lymphocyte response of all treatment groups and by induced differentiation of CD4+ T cells, especially Th17 cells. Additionally, the Lmdd-MPFG vaccine caused maturation of DCs *in vitro*. We demonstrated the synergistic effect of TLR4 and NLRP3 or NOD1 signaling pathways in LM-induced DC activation. These results suggest that the Lmdd-MPFG vaccine is a feasible strategy for preventing HCC.

## INTRODUCTION

Hepatocellular carcinoma (HCC) is the most common type of primary human liver cancer, which is the third leading cause of cancer death worldwide. It is an advanced stage of severe viral hepatitis B or C infection (HBV or HCV) as well as alcoholic liver disease [1, 2]. Unfortunately, many patients with early stage diseases are asymptomatic, and advanced HCC patients barely respond to regular chemotherapy or radiotherapy. As a result, HCC is frequently diagnosed late and requires costly surgical resection or transplantation [3, 4]. While in medium- and late-stage cancer patients, the rate of surgical resection is usually very low (5% to 10%), and the efficacy is compromised by recurrence and metastasis as well [5-7]. Thus, there is an urgent need to explore more effective methods to prevent or treat HCC.

In recent years, research efforts began to focus on immunotherapy in HCC treatment [4, 8]. Studies have shown that immune function is significantly weakened in HCC patients, mainly due to defective T cell responses and functional defects in antigen presentation [9, 10]. As a result, tumor vaccines, which can both induce specific cytotoxic lymphocyte (CTL) responses to tumor cells and change tumor microenvironments [11], have become a safe and effective anti-tumor treatment after surgery, radiotherapy and chemotherapy.

As an intracellular parasite, Listeria monocytogenes (Lm) is generally considered to be an excellent vector for inducing protective cellular immune responses in tumor immunotherapy [12]. After infection, Lm occurs in the cytoplasm of antigen-presenting cells such as macrophages and dendritic cells, and then induces both a CD8+ T cell and CD4+ T cell response by presenting foreign antigens with major histocompatibility complex (MHC) class I and MHC class II molecules efficiently [11, 13]. At the same time, the safety of using Lm is also a critical consideration. Lmdd (Listeria monocytogenes Δdal Δdat) is a highly attenuated vaccine vector, in which the alanine racemase (dal) and D-amino acid aminotransferase (dat) genes have been deleted. Lmdd exhibits minimal toxicity without D-alanine (D-ala) synthesis capacity but modest immunogenicity [14, 15]. In our previous study, a recombinant HCC vaccine Lmdd-MPFG (LM) was constructed based on Lmdd. This LM recombinant epitope expresses the fusion peptide MPFG (multiple peptide fusing genes), a combination of HCC-related human leukocyte antigen (HLA)-A0201 epitopes, full-length hepatitis B virus (HBV) core protein (HBc), HBV-X protein (HBx)52-60, HBx140-148, alpha-Fetoprotein (AFP) 158-166 and melanoma antigen gene-A (MAGE)271-279, and has proven to be effective in inducing strong and specific anti-tumor cellular immunity to HCC [16].

Dendritic cells (DCs) are the most powerful antigen-presenting cells (APCs) and known as important messengers between the innate and the adaptive immune systems [17]. Recent studies show that DCs also play a significant role in tumor immunotherapy. DCs sensitized by tumor-associated antigens or antigenic peptides *in vitro* can induce anti-tumor immune response-specific CTL after transfusion or vaccination in tumor-bearing hosts [18, 19]. It has been reported that Lm can promote the maturation and antigen presentation of DCs and thus effectively stimulate the activation of effector T cells to kill tumor cells [20]. On the other hand, DCs are required for Lm dissemination and proliferation during spleen infection [21, 22]. However, the Lm vaccine utilizes DCs to enhance antitumor immunity, and the probable mechanisms have not been elucidated.

Here, we precisely confirmed that Lmdd-MPFG can be used as an antigen-loading vector to target and promote DC maturation, inducing differentiation of T cell subsets and specific T cell antitumor responses. Our findings revealed that this attenuated Listeria vaccine enhances antitumor activity and DC maturation mainly by modulating CD4+ T cells, especially Th17 cells. Furthermore, we postulated the pattern recognition receptors (PRRs) might be key components in the identification and presentation of Lm by DCs. Several studies have confirmed that Toll-like receptors (TLRs) and NACHT-LRRs (NLRs) act as important sensors for the immune system and are involved in innate effector mechanisms and activation of adaptive immunity [23-25]. This study showed the relevance of interactions between TLR4, NLRP3 and NOD1 in LM-induced DC maturation and anti-tumor responses, which help us understand the immune regulatory mechanisms involved in LM vaccine-related tumor immunotherapy.

## RESULTS

### Lmdd-MPFG can activate BMDCs *in vitro*

**Fig. 1:**
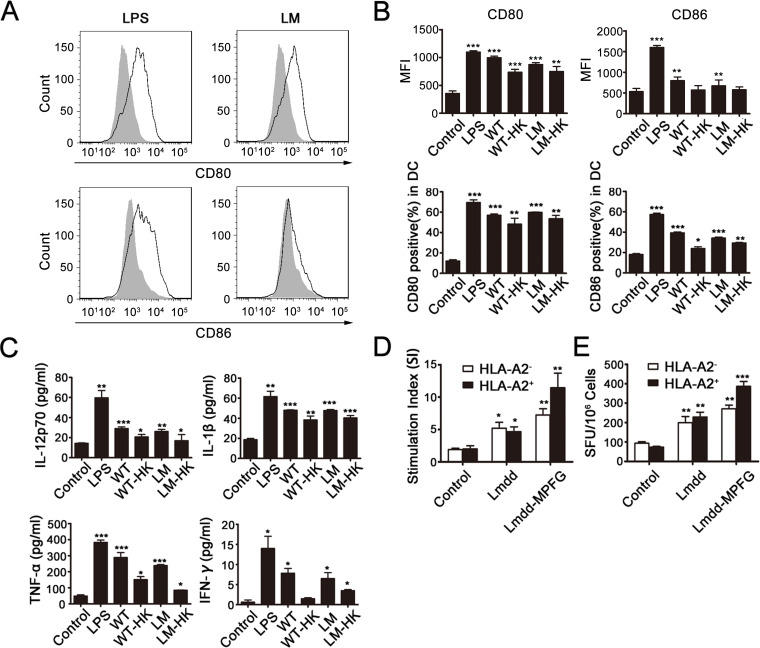
LM promotes maturation of BMDCs and induces MPFG-specific T cells Lmdd-MPFG was compared with WT L. monocytogenes, LPS, and a control (no stimulation), for its potency to promote maturation of BMDCs. DCs were harvested 24 hours after infection with LM (at a MOI of 20), and the expressions of CD80 and CD86 were assessed by flow cytometry, and the peak with gray filled represents the control (A and B). At the same time, supernatants from control or L. monocytogenes–infected DC cultures were collected for measurements of IL-12p70 and TNF-α by ELISA (C). DCs from HLA A2.1+ or HLA A2.1– donors were generated and infected as above, followed by co-culture with allogeneic SPCs for 4 days. Proliferation (D) was measured by ^3^H thymidine incorporation, and stimulation indices (SI) were calculated as described. CD8^+^ TIL function was analyzed by an IFN-γ ELISPOT assay (E). Standard assays were then developed according to the kit manual, and the numbers of spot-forming colonies were calculated. Results were collected from at least three independent experiments. Individual data and mean values are shown. Statistically significant differences are indicated as determined by Student's t-test (*p<0.05, **p<0.01, ***p< 0.001).

To observe DC activation after infection by Lmdd-MPFG, mouse BMDCs were extracted and purified from HLA-A2.1 transgenic mice. Immature DCs were collected and stimulated by LPS, wild type Lm (WT), Lmdd-MPFG (LM), and heat-killed LM (LM-HK) or WT (WT-HK) at a MOI of 20. Twenty-four hours after stimulation, expression levels of the DC surface functional markers CD80 and CD86 were detected by flow cytometry, gated on CD11c (Fig. 1A). The percentage of positively stained CD80 and CD86 cells and their mean fluorescence densities are depicted in histograms shown in Fig. 1B. We found that expression of CD80 and CD86 in DCs was significantly increased in LPS- and LM-stimulated DC groups compared to the expression in the control group. There was no significant difference in CD80 or CD86 expression between Lmdd-MPFG and WT Lm-treated groups.

At the same time, cell culture supernatants for each group were collected after stimulation, and secretion of several cytokines was tested by ELISA (Fig. 1C). Secretion of the cytokines IL-1β and IL-12p70, which are secreted by immunomodulatory DCs, was increased after stimulation, especially the secretion of IL-1β. Although the overall secretion of IFN-γ is low, significant differences were observed. TNF-α and IFN-γ secretion was increased in LPS- and LM-infected DCs compared to the control group.

To explore whether DCs could induce or enhance specific T cell responses via LM stimulation especially with the HLA-A2.1 limiting condition, DCs were generated from the HLA-A2.1 transgenic mice, compared to normal mice, cultured with allogeneic naive splenocytes for 4 days. Then, T-cell proliferation was measured by incorporation of radioactive thymidine during the last 16 hours (Fig. 1D). The result showed that DCs infected with the attenuated strain induced proliferation of allogeneic T cells from HLA-A2.1 transgenic mice compared to control DCs and that T cells showed stronger reactivity to LM when co-cultured with DCs. Additionally, in the IFN-γ ELISPOT assay (Fig. 1E), significantly more spot-forming colonies were observed in the Lmdd-MPFG group than in the controls.

In accordance with these findings, we confirmed that the ability of Lmdd-MPFG to induce DC maturation was not impaired by foreign transgenes and that the modified Lm gene was comparable to wild type Lm. We also showed that Lmdd-MPFG can induce specific T-cell responses in HLA-A2.1 mice.

### NLRs and TLRs and signaling pathways in LM-activated DC maturation

**Fig. 2:**
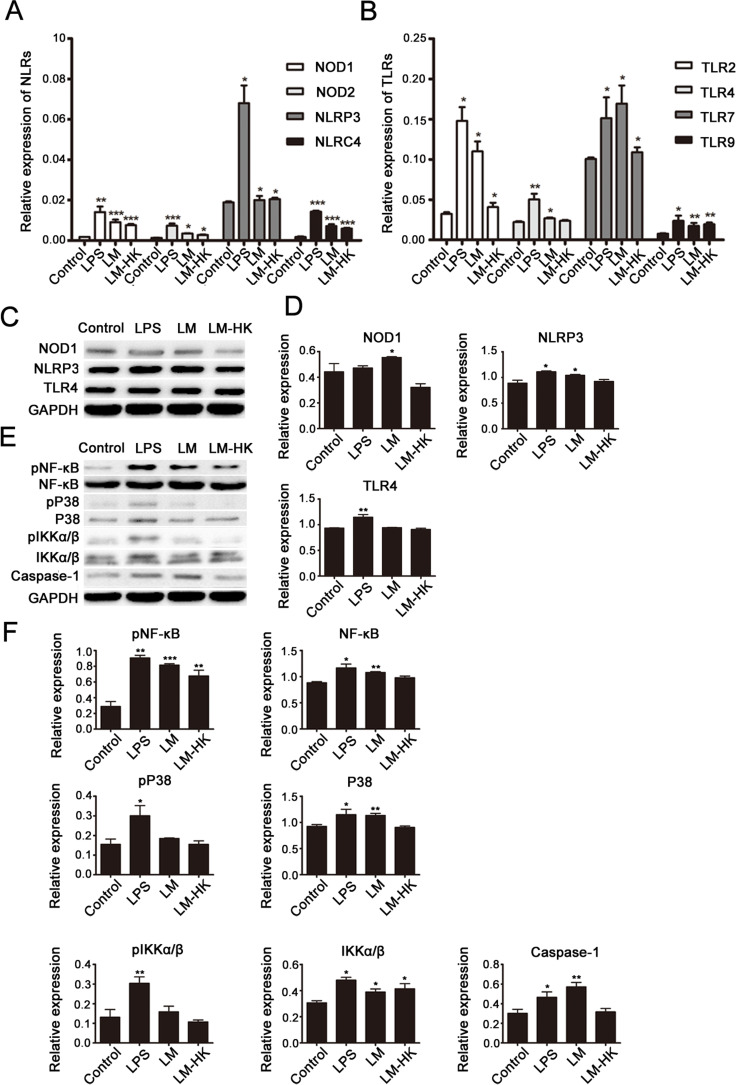
TLRs and NLRs in LM-promoted dendritic maturation BMDCs were collected for 24 h treatment (control, LPS, LM, and LM-HK). Messenger RNA levels of NLRs (A) and TLRs (B) were detected in each group by quantitative real-time PCR. Protein extracts were prepared and relative NLR and TLR protein levels were detected by western blot assays (C). Densitometry values relative to internal controls are displayed in the histograms (D). Activation of several signaling pathways was also analyzed by western blot (E). Summary statistics are depicted in the histogram (F). All data are presented as the mean ± SEM (*p<0.05, **p<0.01, ***p< 0.001).

To investigate relevant receptors involved in LM-induced DC activation, the mRNA expression levels of possible NLRs (Fig. 2A) and TLRs (Fig. 2B) in each DC-treated group (control, LPS, LM, and LM-HK) were detected. Real-time PCR results showed increased expression of NLRs and TLRs in LM-infected groups. An increase in NLRP3 expression was detected only in the LPS-treated group, suggesting a certain connection between NLRP3 and TLR4. Previous studies have shown that the NOD1/NOD2 pathway has a synergistic effect with NLRP3, so we further detected the protein expression levels of NOD1, NLRP3 and TLR in treated DCs by western blotting, (Fig. 2C and 2D). Western blot analyses revealed significant up-regulation of NOD1, NLRP3 and TLR4 in LM-infected DCs and LPS-stimulated DCs compared to the control group.

Emerging lines of evidence suggest that TLR- and NLR-mediated priming signals are required in the Lm infection process [51]. To explore the signaling pathways downstream of the above receptors, we detected the expression levels of NF-kB, MAPK, IKK-α/β and caspase-1 in LM-infected DCs. The histogram in Fig. 2E shows the distribution of expression levels in each group compared to internal controls (Fig. 2F). As shown in the western blotting assay, DCs infected with LM showed effective activation of the NF-kB / pNF-kB signaling pathway, as well as caspase-1 expression. On the other hand, stimulation with LM did not lead to activation of the MAPK or IKK-α/βpathways.

### Interaction of NOD1/NOD2, NLRP3 and TLR4 in BMDCs after LM infection

**Fig. 3:**
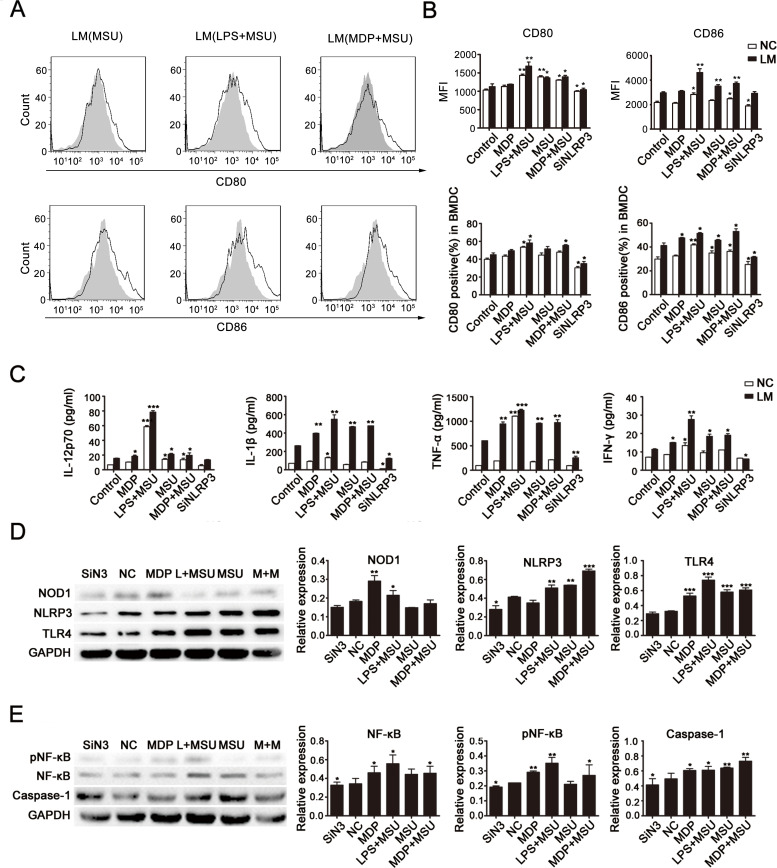
Cross-presentation of NLRs and TLRs in LM-treated DCs DCs were harvested after 24 hours of LM infection (at a MOI of 10) (or of incubation without LM) and then treated to silence or stimulate NLRP3 expression with corresponding receptor agonists (LPS, TLR4 agonist; MDP, NOD1/2 agonist; MSU, NLRP3 agonist) for 6-8 h as described in the Materials and Methods section. Expression of the functional DC molecules CD80 and CD86 was assessed by flow cytometry, and the peak with gray filled represents the control (A and B). Supernatants were collected for quantification of IL-12p70, IL-β, TNF-αand IFN-γ by ELISA (C). Protein levels of NOD1, NLRP3 and TLR4 (D) and expression of NF-kB and caspase-1 (E) were detected by western blot. GAPDH was used as the internal control, and summary statistics are depicted in the histograms. Each data point represents the mean ± SEM from three independent experiments (*p<0.05, **p<0.01, ***p< 0.001).

To further confirm the effect on NLRP3 expression and the interactions between the NLRP3, NOD1/2 and TLR4 signaling pathways in the LM-induced DC-activation process, we collected immature DCs and subjected them to different treatments (control, MDP, LPS+MSU, MSU, MDP+MSU, SiNLRP3). NLRP3 expression was silenced in immature DCs with or without LM infection. After stimulation by corresponding agonists (LPS, TLR4 agonist; MDP, NOD1/2 agonist; and MSU, NLRP3 agonist) for 6-8 h, DC functional phenotypes were detected by flow cytometry (Fig. 3A and 3B). We found that both the CD80 and CD86 expression levels were down-regulated in SiNLRP3 DCs compared to the control, which indicated an important role for NLRP3 in DC activation. Without LM stimulation, CD80 and CD86 levels were increased in the MSU-treated group, and a higher expression occurred with MSU treatment combined with LPS and MDP. More significant changes were observed in the LM-stimulated groups; the combination of agonists led to significant up-regulation in LM-induced DC maturation.

Further, after stimulation by agonists, we detected the expression of cytokines in the supernatants of each group by ELISA (Fig. 3C). The results indicated significantly higher secretions of IL-12p70, IL-1β, TNF-α, and IFN-γ in the LM treatment groups compared to those not treated with LM. Additionally, the secretion levels of these cytokines were much greater after LPS+MSU stimulations but were decreased in the SiNLRP3 group.

Finally, we examined the protein expression of these receptors in cells of each group by western blot analysis. As shown in Fig. 3D, in LM-infected groups, the expression of NOD1, NLRP3 and TLR4 were increased in DCs after LPS+MSU treatment but decreased in the SiNLRP3 group compared to the control group. We further investigated the signaling pathway and found that the LPS+MSU and MDP+MSU stimulations induced a significant activation of the NF-kB pathway and of caspase-1 (Fig 3E). These data confirmed the essential function of NLRP3 and its crosstalk with NOD1/NOD2 and TLR4 in LM-induced DC presentation.

### DC-promoted LM-mediated antitumor effect *in vivo*

**Fig. 4:**
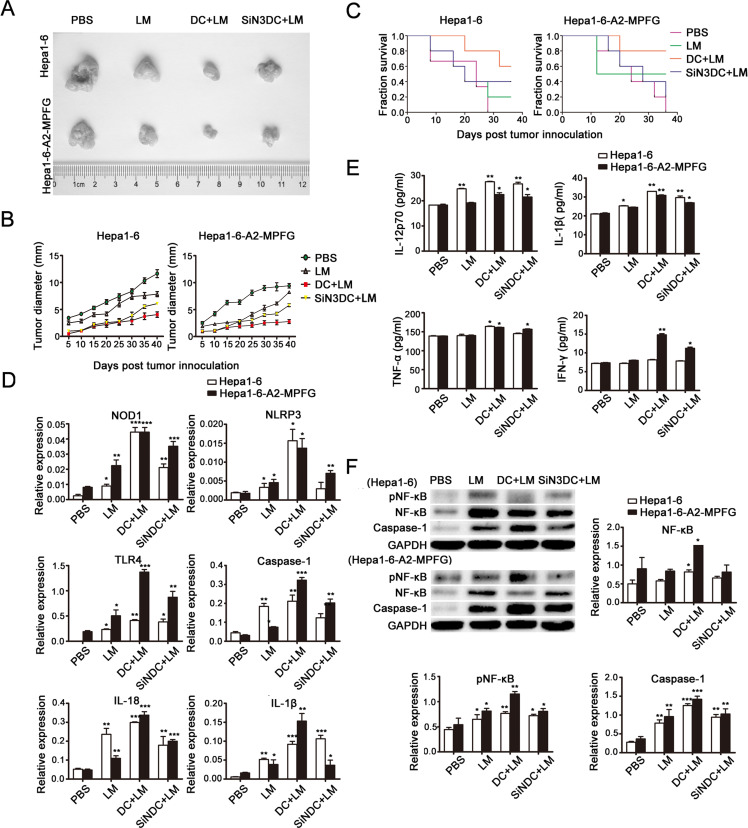
Combined treatment with DCs and LM immunization plays a role in tumor suppression *in vivo* Subcutaneous tumors were generated by injecting mice with Hepa1-6-MPFG tumor cells; corresponding control cells were injected into mice to generate controls. Mice were then immunized with PBS, Lmdd-MPFG, DC+LM or SiNLRP3DC+LM (n=5/group). Tumor sizes were measured every 3 days, and tumors were isolated on day 45. The tumor sizes and weights were measured and compared between groups (A). The tumor growth rates (B) and long-term tumor-free survival times (C) of mice in the vaccination group are shown. Error bars show SEM. Long-term tumor-free survival at day 45. Each data point represents the mean ± SEM from four single tumors. Mouse spleens were isolated, and SPCs were purified and cultured. Quantitative real-time PCR was used to detect expression of NOD1, NLRP3, TLR4 and of the downstream molecules caspase-1, IL-1β and IL-18 in SPCs (D). (E) SPCs were cultured overnight, and then secretion levels of IL-12p70, IL-1β, TNF-α and IFN-γ in the culture supernatant were measured by ELISA. A western blot assay was used to detect the activation of NF-kB and caspase-1 in SPCs (F). GAPDH was used as a loading control. Each data point represents the mean ± SEM from three independent experiments (*p<0.05, **p<0.01, ***p< 0.001).

To establish tumor models, HLA-A2 Tg mice were challenged with 5×10^4^ Hepa1-6 or Hepa1-6-MPFG (co-expressing HLA-A2 and MFPG) tumor cells subcutaneously. Three to seven days after tumor inoculation, the tumor-bearing mice were vaccinated with PBS, Lmdd-MPFG, DC+LM or SiNLRP3DC+LM (n=5/group) at weekly intervals. To evaluate the anti-tumor effects of LM-infected DCs, we measured the tumor sizes (Fig. 4A) and noted the growth rates of tumors in each group (Fig. 4B). The survival rates were also examined (Fig. 4C). Immunization with LM inhibited the development and delayed the growth rates of tumors, and tumor suppression was more effective after intravenous injection of DCs infected by LM. The survival rate of the infected DC-reinfused mice was also the highest of all the groups. The tumors were isolated from tumor-bearing mice at day 45, and SPCs were extracted. Messenger RNA and protein expression levels of NLR, TLR and their downstream products were detected by real-time PCR and western blot, as shown in Fig. 4D and Fig. 4F, respectively. Significant up-regulation of expression of these molecules in the DC+LM group was observed. Further, these SPCs were cultured for 24 h *in vitro*, then the cell culture supernatants were collected, and secretion of cytokines was measured by ELISA (Fig. 4E). Among these cytokines, we found a particular increase in IFN-γ in DC+LM immunized SPCs, which indicated that effective tumor suppression could be achieved by combined DC and LM function.

### DCs are required for specific LM-mediated tumor suppression in the spleen

**Fig. 5:**
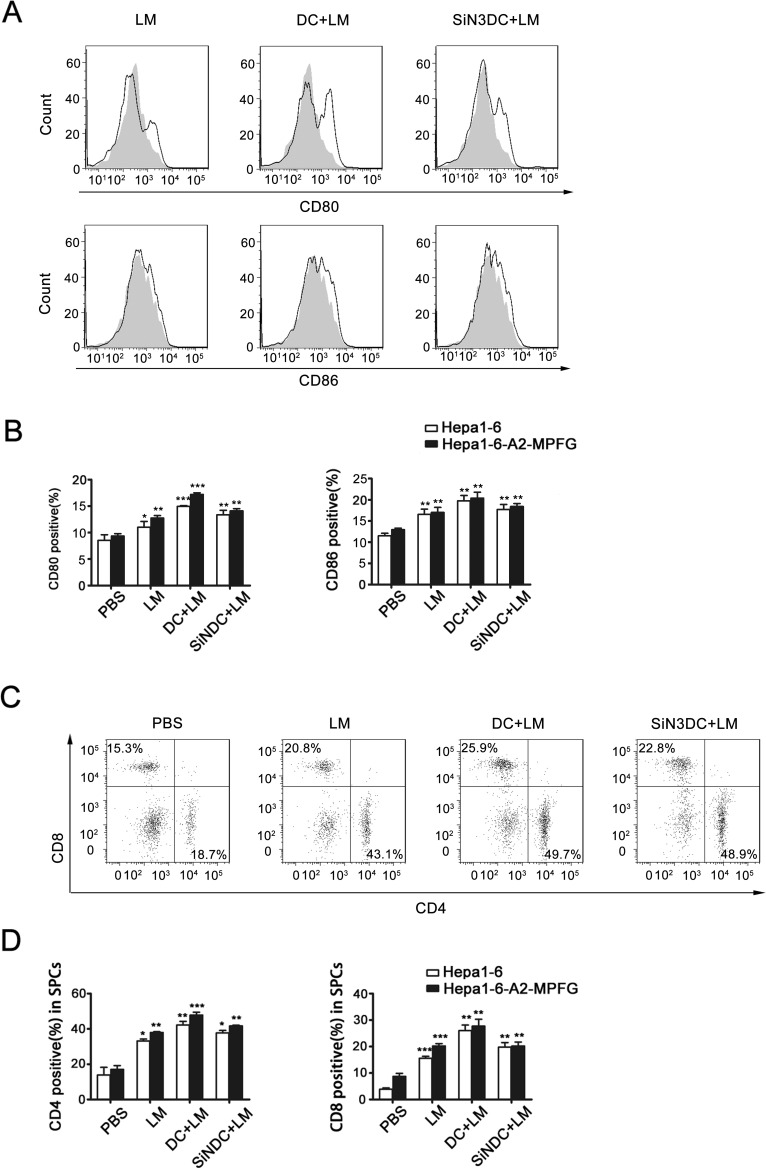
Combined DC and LM treatment increases immune system effects in spleen SPCs were extracted from tumor-bearing mice of each vaccination groups (n=5), pooled and stained for DC or T cell markers. CD80 and CD86 expression and frequency of mature DCs in the SPCs were analyzed by flow cytometric analysis of cells from the vaccinated or control group, and the peak with gray filled represents the PBS group as control (A and B). CD4+ and CD8+ T cells in SPCs were detected by flow cytometric analysis, and the frequencies of each were analyzed in histograms (C and D). Each data point represents the mean ± SEM from at least three independent experiments (*p<0.05, **p<0.01, ***p< 0.001).

To explore the DC-mediated effects under LM infection *in vivo*, we obtained mouse spleen mononuclear lymphocytes (SPCs) from each group as described above. Functional DCs were detected by flow cytometry (Fig. 5A and Fig. 5B). In the DC+LM group, the percentage of functional DCs in spleen was increased compared to the percentages in other groups. In addition, we examined the percentages of CD4+ and CD8+ T cells induced in spleen mononuclear lymphocytes. As indicated by the data shown in Fig. 5C and Fig. 5D, in the spleen, either the percentage of CD4+T or CD8+T cells was up-regulated, especially CD4+ T cells. Thus, we speculate that DCs are necessary to improve LM immunity in the spleen.

### DCs enhance CTL responses against LM infection and induce anti-tumor effects

**Fig. 6:**
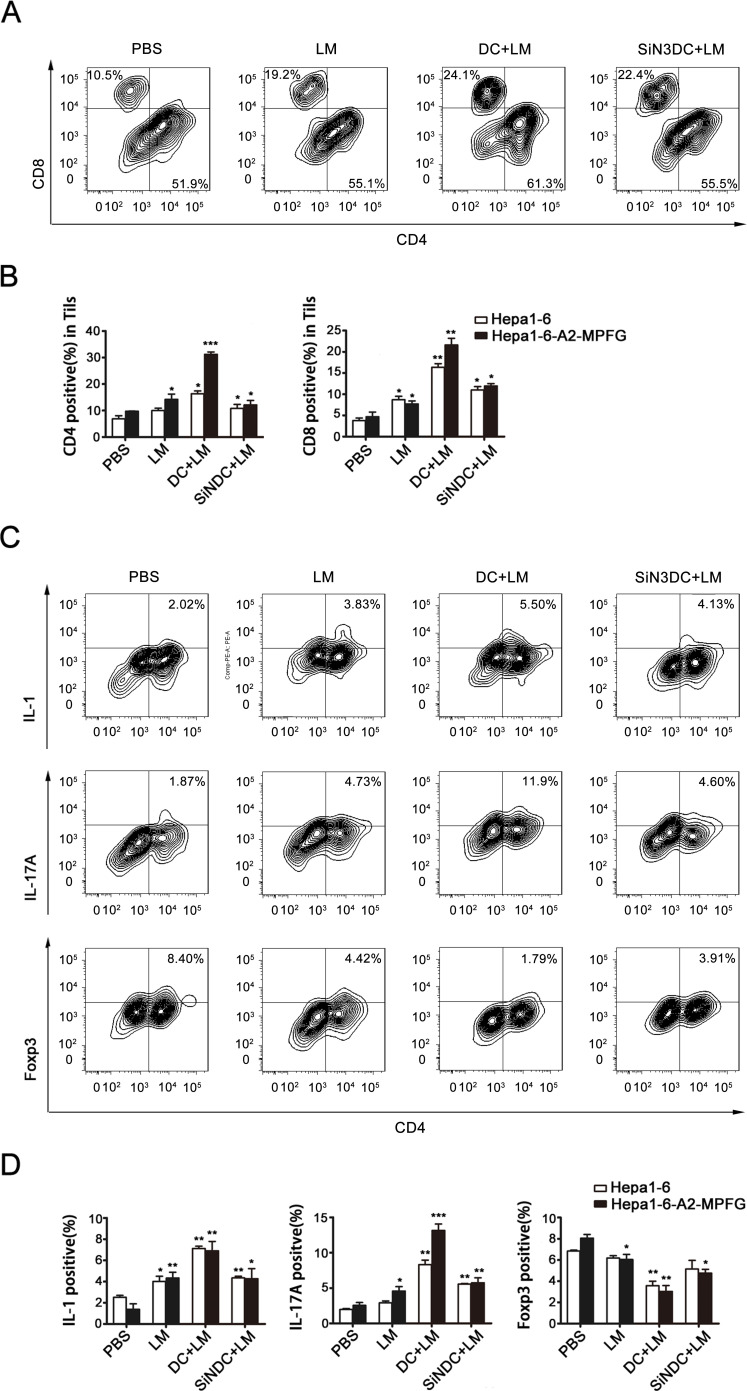
Combination therapy increases the number of Th17 cells within the tumor TILs were separated from the treated mice in each group. CD4+ and CD8+ expression in TILs were evaluated by flow cytometry (A and B). CD4+ T cell differentiation was observed, TILs were stained with antibodies to detect IL-1- and IL-17A-producing cells and Treg cells by flow cytometry (C and D). Foxp3 was detected using intracellular antibody staining. Representative flow cytometry dot plots from each group show Foxp3 expression. Cumulative data for the percentage of positive cells in the gated CD4+ T cell subsets of TILs are illustrated in the dot plots. Data were analyzed in histograms of at least three independent tests and then analyzed by Student's t-test (*p<0.05, **p<0.01, ***p< 0.001).

Tumor-infiltrating lymphocytes (TILs) were isolated from treated mice from each group. We first examined the CD4+ and CD8+ T cell populations among the TILs to evaluate the specific T-cell response. As shown in Fig. 6A, the numbers of CD4+ and CD8+ T cells were significantly increased in vaccinated mice. Moreover, the numbers of CD8+ T cells in combination therapy mice were particularly increased compared to that of mice in other immunization groups.

Next, to explore the regulatory functions of DCs in the LM-immunized tumor microenvironment, CD4+ T cell differentiation was investigated. The percentages of Th1, Th17 and Treg cells were determined by flow cytometry and are shown in Fig. 6B, Fig. 6C and Fig. 6D, respectively. The percentage of Treg cells was reduced in the treated groups, Th1 and Th17 cell percentages were increased in LM-treated mice, and the percentage of IL-17A-producing T cells was significantly increased in LM+DC mice. From these results, we concluded that DC and LM vaccine combination therapy is effective to induce anti-HCC responses.

### Dendritic cells also show functional defects in HCC patients

**Fig 7:**
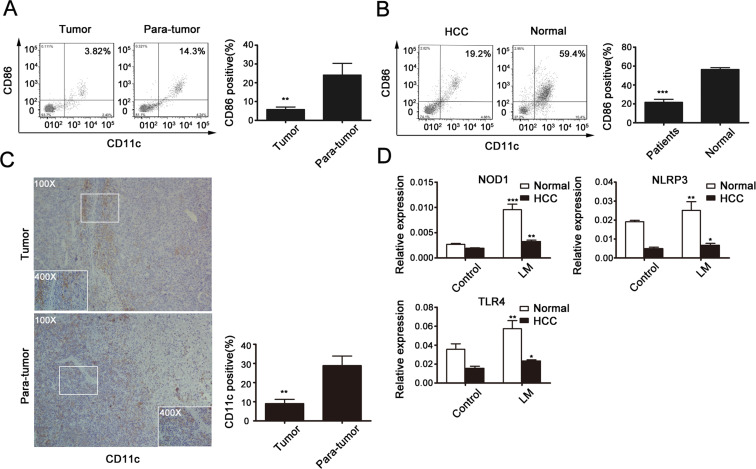
DCs with immature phenotypes and functional defects in HCC patients (A) TILs were isolated from 10 HCC tissues with paired adjacent tissues, and expression of CD80 and CD86 was assessed by flow cytometry. (B) Peripheral blood DCs were obtained from 10 HCC patients and 3 normal people. Proportions of functional DCs were detected by flow cytometry. (C) IHC staining of CD11c in human HCC and adjacent tissues. Statistical analysis of positive cells per field of each index of the slides stained with CD11c is shown in the histogram. Original magnification, 100×. Data presented are from at least five independent slides. (D) Peripheral blood DCs were infected by LM at a MOI of 20; real-time PCR was used to determine the expression of NOD1, NLRP 3 and TLR4 in HCC or normal peripheral blood DCs. All data represent the mean ± SEM from at least three independent experiments (*p<0.05, **p<0.01, ***p< 0.001).

Functional molecules expressed by DCs were detected in the cancer and adjacent tissues of HCC patients (Fig. 7A) as well as in peripheral blood of patients or normal individuals (Fig. 7B). DCs in tumor samples and in HCC patients' peripheral blood showed functional defects compared to those in noncancerous tissues and samples from normal people. To verify the deficiency of DCs in HCC, we further examined the expression of CD11c in paraffin-embedded HCC samples by immunohistochemical analysis (Fig. 7C). As shown, weak cytoplasm staining and poor distribution of CD11c was exhibited in HCC samples, while non-cancerous tissues showed stronger expression, especially in areas of inflammation.

Finally, in order to explore the role of LM and LM-induced responses in HCC patients and in normal people, peripheral blood DCs were obtained and infected by LM, then total RNA samples were extracted, and NOD1, NLRP3 and TLR4 expression levels were analyzed by real-time PCR (Fig. 7D). The expression levels of these receptors, especially of NLRP3, were decreased in the LM-infected group. These data suggest that DCs and NLRP3 might have an important function in the LM vaccination-mediated suppression of HCC.

## DISCUSSION

Hepatocellular carcinoma (HCC) is the fifth most common and lethal malignant cancer worldwide, and its incidence is increasing. The general etiologies of HCC are hepatitis B and C viral infections and intensive alcohol consumption [10, 26]. Current treatment options include liver transplantation, surgical resection and local ablative therapies. However, curative resection is not possible for the many patients with poor hepatic reserve or multiple tumors, and the recurrence rate remains high after tumor resection [19, 27, 28]. There is a pressing need for novel strategies to combat the development and recurrence of hepatocellular carcinoma (HCC). Immunotherapy represents a potential therapeutic option for patients with hepatocellular carcinoma (HCC). It could induce specific self-regulation of the immune system to find and suppress tumor cells.

Listeria monocytogenes (Lm) is an intracellular bacterium that has a natural capacity to elicit potent innate and antigen-specific cellular immune responses. These biological properties make L. monocytogenes a promising platform for the development of anti-cancer therapy [29-33]. Several preclinical studies have demonstrated the safety and efficacy of attenuated L. monocytogenes strains in presenting tumor-associated antigens to the immune system for the clinical treatment of various cancers [34-41]. Here, we focus on the features of an attenuated hepatocellular carcinoma-specific Listeria vaccine Lmdd-MPFG (LM) in the context of HCC immunotherapy.

The stable fusion of HCC-associated antigen genes into the attenuated L. monocytogenes Lmdd makes it potent and safe to induce specific antitumor cellular immunity to HCC [16]. Studies have shown that wild type (WT) Lm and the attenuated strains, when administered intravenously *in vivo*, are rapidly taken up by APCs, particularly DCs in the spleen and the liver [30, 31]. As the classical APC, DCs have been shown to play a role in the containment of Lm during early stages of infection [22]; moreover, they have been shown to be essential for the priming of adaptive immune responses to inhibit tumor development [21, 42-44]. In our study, we observed the significance of LM infection in DC activation and examined the functional phenotype of the activated DCs. We found clear up-regulation of CD80 or CD86 expression in DCs, as well as increased secretion of cytokines from infected DCs. Therefore, LM is highly effective in stimulating the maturation of DCs, which then efficiently cross-present antigens to activate CD8+ T cells.

Studies have found that DCs in HCC patients have an immature phenotype and are functionally defective; and DCs are also considered important in many other cancer prevention processes [45-48]. We therefore proposed a strategy for HCC treatment by combining DCs and an LM vaccine. In tumor-bearing mice, LM immunization delayed the growth of tumors, and the tumor suppression was much more evident after intravenous injection of DCs infected by LM. The survival rate of DC-reinfused mice was also the highest among all treatment groups. Moreover, DCs function to induce specific CTL responses or to change the expression profiles of CD4+ T cells to enhance specific immunomodulatory effects [18, 22, 42, 49, 50]. Thus, in the in vivo study, we examined the function of DCs in mouse spleens and detected CD4+ and CD8+ T cells in both spleen and tumor tissues. We found that in the group treated with a combination of the LM vaccine and DCs, the percentage of functional DCs in spleen was increased compared to other groups of immunized mice. In the spleen, the percentages of CD4+ and CD8+ T cells were increased, especially of CD4+ T cells. Among the TILs, the presence of CD4+ T cells or CD8+ T cells was increased in vaccinated mice, and the growth of CD8+ T cells in combination therapy mice was distinct from other mice in single treatment groups. Furthermore, the CD4+ T cell subsets among the TILs were examined to gain insight into immune regulation in the tumor microenvironment. We found that Treg cells were less abundant, while the percentages of Th1 and Th17 cells were increased in LM-treated mice. Interestingly, the percentage of IL-17A-producing T cells was significantly increased, especially in LM and DC co-treated mice. From these results, we conclude that DC and LM vaccine combination therapy is a promising innovation for inducing anti-HCC responses and that perhaps tumor suppression is mainly enhanced by the production of T17 cells. But further understanding of the mechanisms involved is needed to confirm the efficacy of this treatment.

Toll-like receptors (TLRs) and nucleotide-binding oligomerization domain-like receptors (NLRs) serve as pattern recognition receptors (PRRs) that recognize different but overlapping microbial components. They provide the first line of protection against invading microbial pathogens and are mediated by phagocytes such as macrophages and DCs [51-53]. It is important to explore the role of TLRs and NLRs in LM-induced DC maturation. Many studies have shown that the NOD-like receptor (NLR) family pyrin domain-containing 3 (NLRP3) inflammasome is essential for activating DCs in response to Lm infection and for inducing secretion of the inflammatory cytokines interleukin (IL)-1β and IL-18 by activating caspase-1 [54-57]. Furthermore, more and more studies have found that the presence of interaction between TLRs and NLRs could indicate an efficacious co-presentation of antigen to initiate immune responses [58-61]. Here, we investigated the role of related molecules in LM-induced DC activation, particularly NLRP3, by comparing LM- and LPS-treated mice. The results suggest a certain relationship between NLRP3 and TLR4. Previous studies have shown that the NOD1/NOD2 pathway has a synergistic effect with NLRP3 [58]. Consistent with those results, we also observed significant up-regulation of NOD1, NLRP3 and TLR4 in DCs infected with LM compared to DCs from the control group. Furthermore, NLRP3 expression in immature DCs (with or without LM infection) was silenced or stimulated by corresponding agonists *in vitro*. Reduction of NLRP3 expression inhibited DC maturation, while up-regulation of NLRs or TLR4 resulted in the opposite. Thus, our findings confirmed that NLRP3 induces effective activation of DCs and that the interaction of NLRP3, NOD1/2 and TLR4 contributes to LM-induced DC antitumor activity mainly by activating the NF-kB signaling pathway. Finally, the results in human samples also verified the findings in mice. Therefore, interactions between NLRP3, NOD1/2 and TLR4 might constitute a potential immune regulation mechanism that accounts for the efficacy of DC and LM combination therapy.

In conclusion, our results revealed the promising therapeutic potential of combining DCs and the specific HCC vaccine Lmdd-MPFG in HCC immunotherapy. We also elucidated the mechanisms contributing to the synergistic effect of the combined treatment. Due to the absence of a systemic therapeutic treatment, this combination immunotherapy represents a potentially beneficial option for HCC patients. We also expect that this DC therapeutic regimen can be applied to other types of cancer that have been shown to respond to Lm-based vaccines.

## MATERIALS AND METHODS

### Preparation and stimulation of BMDCs

Mouse BMDCs were prepared as previously described [16]. DCs were used for experiments after 7-8 d of culture, at which point CD11c expression was analyzed by flow cytometry. The Listeria strain has been described in detail previously. Bacterial strains (Wild type Lm and Lmdd-MPFG) were cultured in BHI medium and concentrations were determined during the bacterial logarithmic growth phase (OD600=0.6-0.8). Heat-inactivated bacteria (WT-HK and LM-HK) were prepared by incubating at 80°C for 2 hours. Immature DCs were infected by bacteria at a MOI of 20. After a 2-4 h infection, bacteria were removed by incubating DCs in RPMI1640 containing 40 μg/ml gentamicin (Sigma, St Louis, MO, USA).

BMDCs were stimulated with LPS (1 μg/ml), MDP (10 μg/ml), MSU (250 μg/ml), LPS and MSU together, or MDP and MSU together for 6-8 h. LPS, MDP, MSU were purchased from Sigma Chemical Co. (Sigma, St Louis, MO, USA). DCs were transfected with control NC siRNA or siRNA to NLRP3, and the siRNA sequences were: NC: sense: 5′-AATTCTCCGAACGTGTCACGT-3′, antisense: 3′-ACGTGACACGTTCGGAGAATT-5′; NALP3/NLRP3: sense: 5′-CACGCTAATGATCGACTTCAA-3′, antisense: 3′-TTGAAGTCGATCATTAGCGTG-5′.

### Isolation of tumor-infiltrating lymphocytes (TILs) and SPCs

Tumors and spleens were removed from mice or tissues from HCC patients, and single-cell suspensions were prepared by enzymatic digestion. Resected tumors were weighed, minced into small (1–2 mm^3^) pieces with a scalpel, and immersed in 10 mL of digestion mixture (5% FBS in RPMI 1640, 0.5 mg/ml collagenase A (Roche Diagnostic), 0.2 mg/ml hyaluronidase, type V (Sigma-Aldrich), and 0.02 mg/ml DNase I (Sigma-Aldrich)) per 0.25 g of tumor tissue. The resulting cell suspensions were filtered sequentially through 70- and 40-μm cell strainers (BD Falcon) and washed with 5% FBS in RPMI 1640. Red blood cells (RBC) were lysed by brief incubation in 0.15 M ammonium chloride solution, and cell debris was removed by centrifugation using a lymphocyte isolation sterile solution (Ficoll-Paque^TM^ PLUS) as recommended by the manufacturer (GE Healthcare Bio-Science AB).

### Cell staining and flow cytometry

After stimulation, mouse BMDCs were harvested and labeled with PE-Cy5.5–conjugated mAb against CD11c (eBioscience, San Diego, CA, USA), PE-conjugated anti-CD80 antibody (eBioscience, San Diego, CA, USA), and APC-conjugated anti-CD86 antibody (eBioscience, San Diego, CA, USA). Human TILs and PMDCs were isolated and stained with anti-CD11c-FITC and anti-CD80-PE. After isolation of TIL populations and SPCs from tumor-bearing mice, cells were stained with CD3-PE, CD4-FITC, CD8-APC, IL-1-PE, IL-17A-PE and Foxp3-PE (eBioscience, San Diego, CA, USA). Antibodies and their respective isotypes, used as negative controls for surface and intracellular staining, were all purchased from BD PharMingen. The mouse regulatory T cell staining kit (eBioscience) was used for intracellular cell staining for Foxp3 according to the manufacturer's instructions. Data were collected using a FACSCalibur flow cytometer (BD Biosciences, San Jose, CA, USA) and analyzed with FlowJo software (Tree Star, Ashland, OR, USA).

### T-cell proliferation assay

BMDCs and naive T cells were isolated from autologous mice and were co-cultured at a 1:20 ratio stimulated with or without LM in the presence of IL-2 (20 U/mL) (Protech, London, UK). Then, 200 μl of the final culture volume was incubated at 37°C for 5 days and pulsed with [3H] thymidine at 0.4 μCi/well 16 h before harvesting. Proliferation was measured using a Wallac 1450 scintillation counter. A liquid flash counter was used to detect CPM values; results were averaged from readings from 3 holes and expressed using the stimulation index: SI = (cpminfected - cpmmedium)/(cpmuninfected - cpmmedium).

### Interferon-γ ELISPOT assay

Antigen-specific IFN-γ-secreting T cells from naive T cells promoted by LM-stimulated DCs were detected using ELISPOT kits (BioSource International, Camarillo, CA) as previously described [14]. Briefly, the above co-cultured cells were added to each well (10^7^ cells/well) and incubated with IL-2 (5 U/ml) overnight at 37°C. IFN-γ spots in the wells were then developed according to the manufacturer's instructions. Results were expressed as spot-forming units (SFU)/10^6^ cells after subtracting background spots.

### Cytokine analysis

Samples from supernatants were collected and tested for the presence of cytokines (IL-1beta, IL-12p70, TNF-a, IFN-r) using corresponding mouse ELISA kits (Dakewe Biotech, China) according to the manufacturer's recommendations.

### Quantitative RT-PCR analysis

Total RNA was isolated with Trizol reagent (Invitrogen, USA) according to the manufacturer's instructions. The extracted RNA was dissolved in DEPC-treated ddH2O and subjected to DNase I treatment (Fisher Scientific, USA) to avoid genomic DNA contamination. One microgram of RNA was reverse transcribed to cDNA using the ABI high-capacity cDNA Reverse Transcription Master Kit. For quantitative real-time PCR analysis, cDNA was amplified using the FastStart Universal SYBR Green Master (ROX) Mix (Roche) in a 7500 Fast Real-Time PCR System (ABI). All reactions were performed in triplicate. The 2-ΔΔCT method was used to measure the gene expression (OAZ-1 and GAPDH as the internal control). All primers were designed by Primer Premier 5.0 and synthesized by Integrated DNA Technologies (Coralville, IA).

Sequences of all primer sets are shown in the Table below.

**Table:** 

Primer:
NOD1-F:AGATGGAAGGCACCCCATTG; R:TCTTTCGGACCTTGTCAGGC
NOD2 -F:TTGAGTGTGCTCTTCGCTGT; R:CCCTTATCACCCACGCTGTT
Nlrp3-F:AGCCAGAGTGGAATGACACG; R:CGTGTAGCGACTGTTGAGGT
NLRC4-F:CATTGATGCTGCCTTGGTGC; R:CCGCTAAATCCAACTGCTGC
NLRP6-F:GCTGAAGGGCTCTCAAAGCA; R:TCGGAAAGGTCTCGGCAAAC
GAPDH-F:GCCTCGTCCCGTAGACAAAA; R:GATGGGCTTCCCGTTGATGA
TLR2-F:GGTGCGGACTGTTTCCTTCT; R:TCCTGAGCAGAACAGCGTTT
TLR4-F:TCAGAACTTCAGTGGCTGGAT; R:GTCTCCACAGCCACCAGATTC
TLR5-F:GAATCCCGCTTGGGAGAACA; R:TTCCAAGCGTAGGTGCTCTG
TLR7-F:TCCTCCACCAGACCTCTTGA; R:TCTGTGCAGTCCACGATCAC
TLR9-F:ATAAGGCACAGAGCGCAGTT; R:ATCTCGGTCCTCCAGACACA

### Western blot

Whole cell lysates were prepared in modified RIPA buffer (150 mM, 50 mM Tris-HCl, 50 mM NaF, 5 mM EDTA, 0.5% (wt/vol) sodium deoxycholate and 1% Triton X-100) supplemented with phosphatase and protease inhibitors according to the manufacturer's protocol. Protein concentrations were measured using the BCA assay (Pierce, Rockford, USA). Equal amounts of proteins were mixed with SDS sample buffer, resolved by 10% SDS-polyacrylamide gel electrophoresis (PAGE) and transferred to a polyvinylidene difluoride (PVDF) membrane (Millipore, Eschborn, Germany). The blots were blocked with 5% non-fat dry milk solution for 1 h at room temperature and incubated overnight with primary antibodies at 4°C. The primary antibodies used were rabbit anti-NLRP3, rabbit anti-TLR4, rabbit anti-NOD1, rabbit anti-caspase-1 (Abcam, Cambridge, MA), rabbit anti-P65,pP65,P38,pP38,IKKa/b and pIKKa/b, and anti-GAPDH (Cell Signaling Technology, Boston, USA). The secondary antibodies (Santa Cruz Biotechnology, Santa Cruz, CA) were used at 1:2,000 (v/v) dilutions in PBS + 0.1% Tween 20 for 1 h at room temperature, and immunoreactivity was detected using an ECL kit (Thermo Scientific, Etten-Leur, The Netherlands).

### Mice and tumor cell lines

C57BL/6 mice were bred by and purchased from the Central Animal Lab of Nanjing Medical University. The experiments were conducted when the mice were 6–8 weeks old. All experimental protocols used in this study were approved by the Institutional Ethics Committee of Nanjing Medical University. Breeding pairs of HLA-A2.1 Tg mice with a C57BL/6 background were purchased from the Model Animal Research Center of Nanjing University. Six- to eight-week-old Tg mice were used at the start of the experiments. Hepa1-6, a mouse hepatoma cell line derived from C57BL/6 mice, was obtained from the American Type Culture Collection (Manassas, VA). The cell line Hepa1-6-HLA-A2.1/MPFG stably expresses HLA-A2.1 and is transformed with the MPFG gene. Cells were maintained in Dulbecco's modified Eagle medium supplemented with 10% FBS (Cellgro), 100 U/ml penicillin, and 100 mg/ml streptomycin. All cells were incubated at 37°C in a 5% CO_2_ incubator.

To establish the tumor models used in this study, HLA-A2 Tg mice were challenged with 5×10^4^ Hepa1-6 or Hepa1-6-MPFG (co-expressing HLA-A2 and MFPG) tumor cells subcutaneously. Three to seven days later, the tumor-bearing mice were vaccinated with phosphate-buffered saline, LM, DC+LM or SiNLRP3DC+LM (n=5/group) at weekly intervals for a total of three weeks. Each week, the tumor sizes were measured with a caliper in two dimensions, and the tumor volumes (V) were calculated with the equation V = (L×W2) ×0.5, with L = length and W = width. Mice were killed on day 40. To determine the effect of the vaccine on tumor development, tumors and spleens were isolated for cellular immune response measurements *in vivo*. Each experiment was performed at least twice, and results were essentially similar unless described otherwise.

The animals were housed under specific pathogen-free conditions. All animal protocols were reviewed and approved by the institutional animal care and use committee at UTHSCSA.

### Clinical samples

Human HCC and adjacent tissue or blood samples in this study were obtained from patients who underwent hepatectomy or liver transplantation for treatment of liver cancer in the First Affiliated Hospital of Nanjing Medical University. TILs or DCs were isolated from tissues and blood samples and were cultured for use in subsequent analyses. All HCC patients gave written informed consent on the use of clinical specimens for research purposes. The study was also approved by the institutional ethics committee.

### Immunohistochemistry

Paraffin-embedded specimens were deparaffinized with xylene, rehydrated using graded ethanol, then boiled in sodium citrate-hydrochloric acid buffer in a pressure cooker for 10 minutes, and cooled for 30 minutes at room temperature to expose antigenic epitopes. Then, the samples were blocked with 5% normal goat serum in 1% bovine serum albumin in PBS for 30 minutes at room temperature. The slides were then incubated with polyclonal rabbit anti human-CD11c antibody at a dilution of 1:500 and incubated overnight at room temperature. After being washed thrice with 0.05% Tween 20 in PBS for 10 minutes, slides were stained with secondary antibody for 1 hour and then washed thrice with 0.05% Tween in PBS for 10 minutes again. Slides were then stained with hematoxylin and washed with running water. For immunohistochemical staining, the samples were then processed using the SP immunohistochemical kit (Maixin, Fuzhou, China), and the immunoreactive proteins were detected using a DAB kit (Maixin, Fuzhou, China). After all the stains, slides were dehydrated with ethanol followed by dimethylbenzene. Finally, after being mounted with cover slides, samples were observed under the microscope (Nikon, Eclipse 80i). The number of positive cells per field of each index was obtained by counting five separated fields (×100) with Image-Pro Plus software (v. 5.0).

### Statistical analysis

SPSS software was used for all statistical analyses. Data were presented as the mean ± SEM. A two-tailed Student t-test was used to assess significant differences between control and treatment groups. P<0.05 was considered to indicate statistically significant differences.
