# Correction: The attenuated hepatocellular carcinoma-specific Listeria vaccine Lmdd-MPFG prevents tumor occurrence through immune regulation of dendritic cells

**DOI:** 10.18632/oncotarget.28154

**Published:** 2022-01-31

**Authors:** Xin Wan, Ci Cheng, Zhe Lin, Runqiu Jiang, Wei Zhao, Xin Yan, Junwei Tang, Kun Yao, Beicheng Sun, Yun Chen

**Affiliations:** ^1^Department of Microbiology and Immunology, Nanjing Medical University, Nanjing, Jiangsu Province, China; ^2^Liver Transplantation Center, The First Affiliated Hospital of Nanjing Medical University, Nanjing, Jiangsu Province, China

**This article has been corrected:** Due to errors during figure assembly, the GAPDH blots in Figures 2C and 2E are accidental duplicates of the GAPDH blots in Figure 3D. The proper Figure 2, produced using the original data, is given below. The authors declare that these corrections do not change the results or conclusions of this paper.

Original article: Oncotarget. 2015; 6:8822–8838. 8822-8838. https://doi.org/10.18632/oncotarget.3558

**Figure 2:**
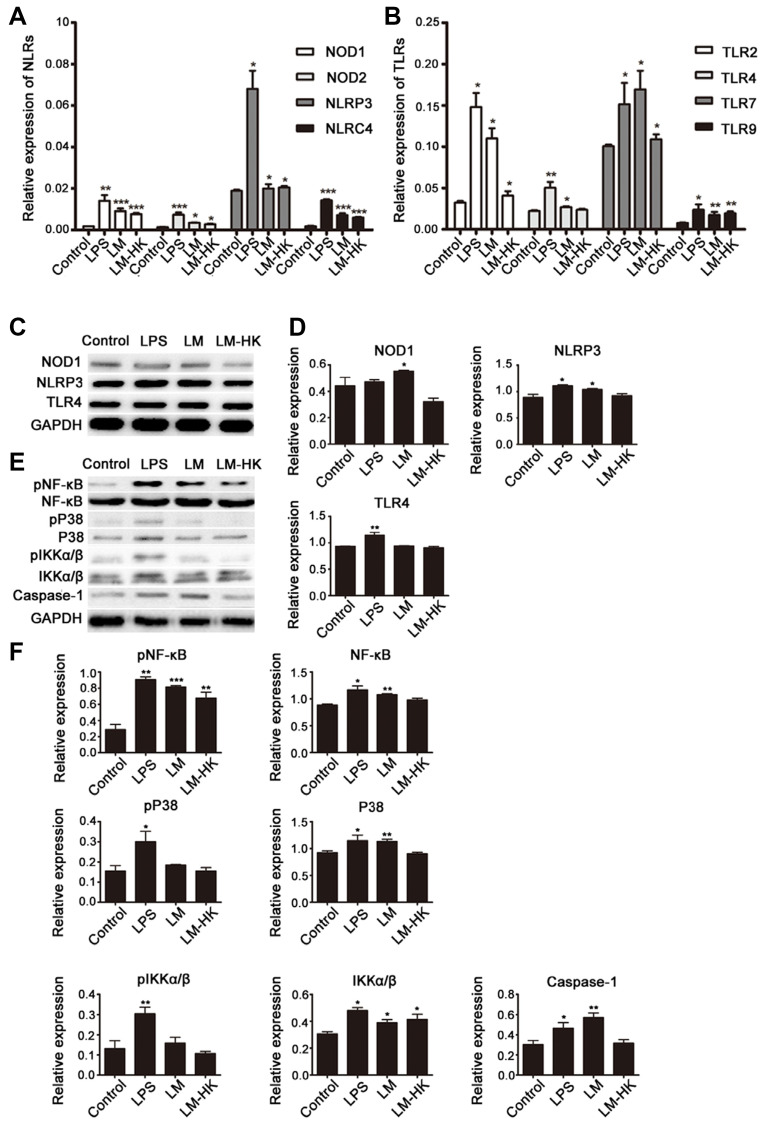
TLRs and NLRs in LM-promoted dendritic maturation. BMDCs were collected for 24 h treatment (control, LPS, LM, and LM-HK). Messenger RNA levels of NLRs (**A**) and TLRs (**B**) were detected in each group by quantitative real-time PCR. Protein extracts were prepared and relative NLR and TLR protein levels were detected by western blot assays (**C**). Densitometry values relative to internal controls are displayed in the histograms (**D**). Activation of several signaling pathways was also analyzed by western blot (**E**). Summary statistics are depicted in the histogram (**F**). All data are presented as the mean ± SEM (^*^ *p* < 0.05, ^**^ *p* < 0.01, ^***^ *p* < 0.001).

